# Strategies for selecting subsets of single-nucleotide polymorphisms to genotype in association studies

**DOI:** 10.1186/1471-2156-6-S1-S72

**Published:** 2005-12-30

**Authors:** Joe M Butler, D Timothy Bishop, Jennifer H Barrett

**Affiliations:** 1 Cancer Research UK Genetic Epidemiology Division, University of Leeds, Cancer Genetics Building, St James's University Hospital, Beckett Street, Leeds LS9 7TF, UK

## Abstract

In genetic association studies, linkage disequilibrium (LD) within a region can be exploited to select a subset of single-nucleotide polymorphisms (SNPs) to genotype with minimal loss of information. A novel entropy-based method for selecting SNPs is proposed and compared to an existing method based on the coefficient of determination (*R*^2^) using simulated data from Genetic Analysis Workshop 14. The effect of the size of the sample used to investigate LD (by estimating haplotype frequencies) and hence select the SNPs is also investigated for both measures. It is found that the novel method and the established method select SNP subsets that do not differ greatly. The entropy-based measure may thus have value because it is easier to compute than *R*^2^. Increasing the sample size used to estimate haplotype frequencies improves the predictive power of the subset of SNPs selected. A smaller subset of SNPs chosen using a large initial sample to estimate LD can in some instances be more informative than a larger subset chosen based on poor estimates of LD (using a small initial sample). An initial sample size of 50 individuals is sufficient in most situations investigated, which involved selection from a set of 7 SNPs, although to select a larger number of SNPs, a larger initial sample size may be required.

## Background

In studies investigating association between disease and candidate genes (or genomic regions), it is inefficient and impractical to genotype every single-nucleotide polymorphism (SNP). Various strategies have been proposed for deciding which subset of SNPs to genotype in a large group of cases and controls with minimal loss of information. Most of these use haplotype frequency estimates derived from a smaller sample of controls, in which all SNPs in the region have been genotyped.

The Genetic Analysis Workshop 14 (GAW14) simulated dataset provided a suitable dataset for the investigation of various aspects of this problem. A number of genomic regions are included in the simulation that exhibit linkage disequilibrium (LD). These regions contain variants associated with disease and have available SNP data.

The aims of this study were: 1) to compare the SNPs selected by 2 different selection strategies and 2) to examine the effect of the size of the initial sample on which all SNPs are genotyped on the choice of subset. Here we report results from the analysis of two regions each containing 7 SNPs.

## Methods

Two loci from the simulated data, D2 and D4, were considered because there was LD within these regions. (The "answers" were known to the authors prior to the study). Data on 5,000 individuals were obtained by using all 100 replicates from the control population, each consisting of 50 subjects.

The pattern of LD within each locus was evaluated by calculating Lewontin's D' coefficient between each pair of SNPs, based on the genotype information, using the *pwld *function in STATA [1]. Seven SNPs from each locus were selected as the starting point for analysis in this study. Because the LD present in the GAW simulated data was not strong, these SNPs were chosen to maximize the LD across the regions covered. For locus D2 there were only 7 SNPs with any D' measure of 0.5 or higher, so these were chosen. For D4 the 7 consecutive SNPs with the highest LD across the region spanned were chosen.

### Selecting an "optimal" subset of SNPs

All 5,000 genotypes from the control population were used to obtain the best possible estimates of haplotype frequencies using SNPHAP [2], which implements a modified expectation maximization (EM) algorithm. Using these haplotype frequencies, subsets of the 7 constituent SNPs can be rated according to some measure of information content. A number of such measures exist, and in this study two are considered:

### Standardized Entropy (S*ε*)

This is a novel measure based on the idea of entropy. A measurement known as entropy (*ε*) has already been considered in the context of measuring LD [3]. Here we propose the use of it for the different, but related, matter of rating a subset of SNPs.

Consider a genomic region made up of *T *diallelic SNPs. Suppose in a sample population, these SNPs construct *N *discrete haplotypes *h*_1_, *h*_2_, ..., *h*_*N*_. Call the population frequencies of these haplotypes *f*_1_, *f*_2_, ..., *f*_*N*_. If only a subset of *s *SNPs (*s *≤ *T*) are genotyped the haplotypes *h*_1_, *h*_2_, ..., *h*_*N *_are partitioned into *M *groups of haplotypes, where *M *≤ *N*. Call these groups *g*_1_, *g*_2_, ..., *g*_*M *_and their respective frequencies *F*_1_, *F*_2_, ..., *F*_*M *_where:



Then entropy is calculated as:



Thus entropy rates highly those subsets that partition the haplotypes into a large number of equally sized groups.

In measuring a single subset, entropy provides little information; it is only really informative when comparing two or more subsets. The entropy measure is therefore standardized by comparing it to the maximum achievable entropy, i.e., the entropy based on all SNPs. In this case all haplotypes are identifiable and hence all "groups" consist of just one haplotype. The S*ε *of a given subset is given by:



### A measure based on the coefficient of determination (R^2^) [4]

This is a measure of how well the SNPs not included in the subset can be predicted from the haplotype groups (*g*_1 _to *g*_*M *_described above) defined by the subset.

Using the haplotype frequencies estimated from the 5,000 individuals, both measures (S*ε *and *R*^2^) were calculated for all subsets of each size (1 to 6). This provides a reference rating for each subset that will be important for evaluating the effect of sample size on SNP subset selection. It also permits the "optimal" subset, the subset with the highest rating, to be identified for each subset size. The "optimal" subsets chosen by the different measures were then compared.

### Effect of sample size on SNP subset selection

The process described in "Selecting an "optimal" subset of SNPs" was repeated, but, instead of using the whole population of 5,000, a random sample of *n *individuals (*n *= 10, 20, 50, 100, 200) was used to estimate the haplotype frequencies. Using these (now less precise) estimates of the haplotype frequencies, all of the SNP subsets were rated by the 2 measures as before, and the optimal subset(s) of each size were identified and recorded. The optimal subset is then assigned its reference rating (see above), i.e., the rating of this subset if "true" haplotype frequencies (estimates based on the total population) were used. If there was more than 1 optimal subset the mean of their reference values was used. This process was carried out for all SNP subset sizes (1 to 6). The whole process was then repeated 100 times for each sample size (*n*) and the mean values recorded.

The rating for a selected subset is maximal if the subset chosen using the sample matches the subset chosen using the whole population to estimate haplotype frequencies. In addition, to give an indication of how effective these selection methods are, a subset of each size was also selected at random and assigned its reference rating.

## Results

**Figure 1:**
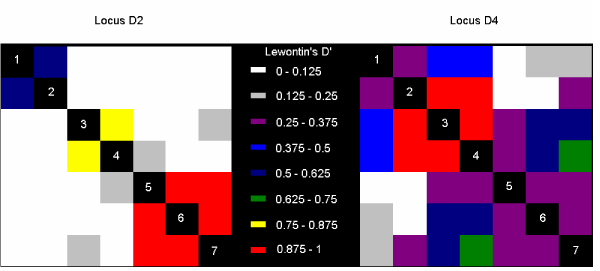
Matrices indicating the extent of LD at loci D2 and D4.

The two matrices in Figure 1 illustrate the degree of LD between the 2 sets of 7 SNPs. LD at D2 is very weak, and most SNPs only show LD with their immediate neighbors. Because of the low LD across the region as a whole, the estimated haplotype frequencies based on all 7 SNPs are all extremely low, the most common haplotype having an estimated frequency of only 8.5%. The LD across locus D4 is higher, although still not strong. The most common haplotype has an estimated frequency of 14.9%. The optimal subsets of each size for each measure are displayed in Tables 1 and 2. The tables also illustrate the degree to which the ratings increase with each increase in subset size. It is of note that there is a high degree of agreement between the subsets selected by the 2 measures, especially for locus D4.

**Figure 2:**
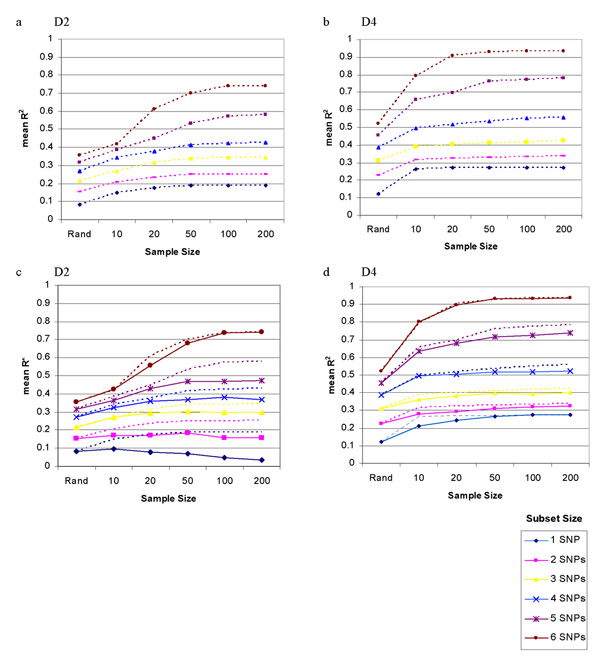
**The effect of sample size on optimal subset selection at D2 and D4 using *R*^2 ^(a and b), and S*ε *and *R*^2 ^(c and d)**. a and b, the optimal subsets are identified from a sample using *R*^2^. They are rated using the *R*^2 ^results obtained from 5,000 individuals. c and d, the optimal subsets are identified from a sample using S*ε*. They are rated using the *R*^2 ^results obtained from 5,000 individuals. The *R*^2 ^graphs are shown as broken lines for comparison.

Figure 2a shows that for locus D2 the rating of the subset selected using *R*^2 ^increases considerably on average as the sample size used to estimate haplotype frequencies is increased, reaching a maximum at a sample size of between 50 and 100. When considering locus D4, with higher LD (Figure 2b), the maximum is reached at a much smaller sample size. Figure 2d demonstrates that the subsets selected by S*ε *are in many cases of similar *R*^2 ^rating to the subsets chosen by *R*^2^, although there are exceptions to this in the D2 locus with very low LD (Figure 2c).

## Conclusion

A new SNP selection method, standardized entropy, has been presented and compared with an existing method, *R*^2^. The subsets identified by the S*ε *measure are similar to those chosen by the *R*^2 ^measure (Tables 1 and 2), rarely differing by more than 1 SNP. Thus S*ε *may be useful in choosing the SNPs to be genotyped, since it is computationally less demanding than *R*^2^. In the analysis of 7 SNPs, computation of *R*^2 ^is 11% slower than that of S*ε*, but the difference is expected to be greater for larger problems, since *R*^2 ^requires a separate computation for each unselected SNP.

For both measures, as expected, the figures show that for any fixed sample size, increasing the number of SNPs in the subset leads to increased information, and for any fixed number of SNPs increasing the sample size used to estimate haplotype frequencies never leads to a decrease in the measure. However, the rate of increase diminishes as sample sizes approach higher values. Thus for all cases considered there is very minimal gain in information in sampling 200 individuals over 100, and in most instances the curve reaches a plateau at a sample size of 50 or less. For both loci the smaller the SNP subset, the smaller the sample size needed to reach the maximum value, although for small subsets the gain in *R*^2 ^over the random baseline value is quite small.

As expected, the measures are generally higher for the D4 locus than for equivalent values at the D2 locus because of the higher degree of LD. For example, the 6-SNP curve attains an *R*^2 ^value of 0.94 for D4 and only 0.74 for D2 and a similar observation holds for all other SNP subset sizes.

One interesting observation from Figure 2a is that the *R*^2 ^value is sometimes higher on average using a smaller subset of SNPs if the estimation is based on a larger sample. For example, at the D2 locus using a 4-SNP subset estimated from 100 controls yields a higher *R*^2 ^on average than a 6-SNP subset estimated from 10 controls.

We have carried out a similar analysis on set of 7 SNPs in the *XPC *DNA repair gene, which are in much higher LD than the GAWdatasets described here. Similar results are observed, although the higher LD results in the corresponding ratings being higher on the *R*^2 ^axis. For these data the lines plateau at a sample size of only around 10, illustrating that the higher the LD the smaller the sample required to select an optimal subset.

To draw more general conclusions this analysis now needs to be repeated using sets of more than 7 SNPs. One problem experienced when assessing datasets containing a large number of SNPs is the heavy computation burden. Current methods use an "exhaustive" search in which all subset combinations are rated. This is not feasible as the number of SNPs increases. In selecting a subset of 15 SNPs from 30 there are 155,177,520 possible combinations to assess. Algorithms that search more efficiently through possible subsets, for example using simulated annealing, are a subject of current research.

A limitation of this analysis to date is that these results do not relate straightforwardly to the power of the subsets selected in detecting disease × gene associations, which is the ultimate goal. A high correlation would be expected between the value of *R*^2 ^and power, but further work is needed to assess directly how the different subset selection methods affect power and in particular to compare subsets selected using S*ε *and *R*^2^.

## Abbreviations

GAW14: Genetic Analysis Workshop 14

LD: Linkage disequilibrium

S*ε*: Standardized entropy

SNP: Single-nucleotide polymorphism

## Authors' contributions

JMB conducted the analyses and jointly authored the paper with JHB, who also supervised and advised on the project. DTB advised on the project and commented on the paper.

## Figures and Tables

**Table 1:** Optimal subsets of locus D2 as identified by the two measures.

No. of SNPs	Standardized entropy								Chapman's *R*^2^							
		
	Optimal subset							Rating	Optimal subset							Rating
1	*1^a^*							0.1932	6							0.189
2	*1*	*5*						0.3848	4	6						0.2553
3	1	4	*5*					0.5718	1	4	6					0.3452
4	1	2	4	*5*				0.7195	1	2	4	6				0.4335
5	1	2	3	4	*5*			0.8353	1	2	3	4	6			0.5794
6	1	2	3	4	5	7		0.9493	1	2	3	4	5	7		0.7399
7	1	2	3	4	5	6	7	1	1	2	3	4	5	6	7	1

^a^SNPs highlighted in red differ between the two uniquely optimal subsets of equal size.

**Table 2:** Optimal subsets of locus D4 as identified by the two measures.

No. of SNPs	Standardized entropy								Chapman's *R*^2^							
		
	Optimal subset							Rating	Optimal subset							Rating
1	3							0.2311	3							0.1539
2	3	5^a^						0.4333	3	6						0.3416
3	3	5	6					0.6021	3	5	6					0.4264
4	1	2	5	6				0.7544	1	3	5	6				0.5652
5	1	3	5	6	7			0.8941	1	3	5	6	7			0.7839
6	1	2	4	5	6	7		0.9827	1	2	4	5	6	7		0.9424
7	1	2	3	4	5	6	7	1	1	2	3	4	5	6	7	1

^a^SNPs highlighted in red differ between the two uniquely optimal subsets of equal size.
